# Low BMI and postoperative outcomes in elderly hip fracture patients: a Japanese nationwide database study

**DOI:** 10.1007/s00774-025-01660-5

**Published:** 2025-11-25

**Authors:** Yu Mori, Kunio Tarasawa, Hidetatsu Tanaka, Ryuichi Kanabuchi, Naoko Mori, Kiyohide Fushimi, Toshimi Aizawa, Kenji Fujimori

**Affiliations:** 1 Department of Orthopaedic Surgery, Tohoku University Graduate School of Medicine, 1-1 Seiryo-machi, Aoba-ku, Sendai, Miyagi 980-8574 Japan; 2 Department of Health Administration and Policy, Tohoku University Graduate School of Medicine, 2-1 Seiryo-machi, Aoba-ku, Sendai, Miyagi 980-8574 Japan; 3 Department of Radiology, Akita University Graduate School of Medicine, 1-1-1 Hondo, Akita, Akita 010-8543 Japan; 4 Department of Health Policy and Informatics, Institute of Science Tokyo, 1-5-45 Yushima, Bunkyo-ku, Tokyo, 113-8519 Japan

**Keywords:** Body mass index, Underweight, Hip fracture, Postoperative complications, Administrative claims database, Osteoporosis

## Abstract

**Introduction:**

Hip fractures are common in elderly individuals and contribute to significant morbidity and mortality. Low body mass index (BMI) is associated with osteoporosis and frailty, yet its impact on postoperative outcomes after hip fracture surgery remains unclear.

**Materials and methods:**

We conducted a retrospective cohort study using Japan’s Diagnosis Procedure Combination (DPC) database. Patients aged ≥ 65 years undergoing surgery for hip fractures from April 2016 to March 2022 were included. Low BMI was defined as < 17.0 kg/m^2^, and < 18.5 kg/m^2^ was used for sensitivity analyses. Patients < 65 years or treated non-operatively were excluded. Primary outcomes included venous thromboembolism (VTE), pneumonia, urinary tract infection, cognitive dysfunction, in-hospital mortality, transfusion volume, and length of stay. Propensity score matching (1:1) was performed, followed by logistic regression.

**Results:**

Of 474,293 patients identified, 63,761 matched pairs were analyzed. Compared to the non-low BMI group, the low BMI group had higher rates of urinary tract infection (3.6% vs. 3.0%; OR 1.203, 95% CI 1.131–1.280), pneumonia (5.3% vs. 3.0%; OR 1.850, 95% CI 1.746–1.961), and in-hospital mortality (3.4% vs. 1.6%; OR 2.233, 95% CI 2.068–2.411). Perioperative transfusion volume was higher in the low BMI group, while VTE was less frequent. Sensitivity analyses using < 18.5 kg/m^2^ confirmed these findings.

**Conclusion:**

Low BMI is associated with increased complications and mortality following hip fracture surgery in the elderly. These findings emphasize the prognostic relevance of BMI in perioperative risk assessment.

**Supplementary Information:**

The online version contains supplementary material available at 10.1007/s00774-025-01660-5.

## Introduction

Hip fractures are among the most common orthopedic injuries in the elderly and are associated with substantial morbidity and mortality [1, 2]. The global annual incidence is projected to reach 4.5 million by 2050 [3], with cases in the United States expected to double from 250,000 in 1990 to 500,000 by 2040 [4]. In Japan, approximately 250,000 proximal femur fractures occur annually, where 13 million individuals are affected by osteoporosis [5, 6]. More than 90% of hip fractures occur in people aged ≥ 65 years, with 30-day and 1-year mortality rates reaching 4.0–5.4% and about 25%, respectively [7, 8].

Individuals with a low body mass index (BMI < 18.5 kg/m^2^) have more than twice the risk of osteoporosis compared with normal-weight individuals [9, 10], and large-scale cohorts confirm low BMI as an independent risk factor for reduced bone mineral density [9, 11]. Proposed mechanisms include diminished mechanical loading on bone, reduced muscle mass, and nutritional deficiencies, all of which contribute to lower peak bone mass and accelerated bone loss [12]. Epidemiological studies further show a strong link between low BMI and osteoporotic fractures, especially hip fractures; for example, postmenopausal women with BMI < 20 kg/m^2^ nearly double their fracture risk [13, 14]. Underweight status not only predisposes to fracture occurrence but is also associated with poorer post-fracture outcomes, including higher mortality and impaired functional recovery [13, 15, 16]. These adverse outcomes are likely attributable to greater frailty, inadequate nutritional reserves, and diminished physiological resilience in individuals who are underweight. Although the World Health Organization defines underweight as a BMI < 18.5 kg/m^2^ [17], several studies in Asian populations and elderly cohorts have indicated that a stricter cutoff of BMI < 17.0 kg/m^2^ better reflects clinically relevant malnutrition and frailty risk [18].

Previous studies utilizing the Diagnosis Procedure Combination (DPC) database in Japan have examined postoperative complication risks in elderly patients with hip fractures and in those undergoing total hip arthroplasty [19–27]. Previous studies from other countries have shown that underweight status is associated with poor surgical outcomes in elderly patients [28–30]. However, the impact of low BMI on postoperative outcomes in elderly hip fracture patients has not been fully clarified. Therefore, the aim of this study was to evaluate the impact of low BMI on postoperative outcomes, including complications, transfusion, and in-hospital mortality, using a large Japanese nationwide database.

## Materials and methods

### Study design

This retrospective cohort study was carried out in accordance with the ethical principles outlined in the Declaration of Helsinki and was approved by the institutional ethics committees.

### Data source

Data for this study were retrospectively obtained from Japan’s nationwide administrative Diagnosis Procedure Combination (DPC) reimbursement database [31]. Upon hospital admission, all patients provided comprehensive informed consent, which included agreement to the proposed treatments and permission for the academic use of their clinical data. The dataset used in this study contains no personally identifiable information. All variables analyzed were extracted from this database. To ensure data integrity and completeness, cases with missing values for any relevant clinical variables were excluded. No imputation techniques were employed; only cases with complete data for all covariates and outcomes were included in the final analysis.

### Inclusion and exclusion criteria

The sample size was determined by the fixed study period rather than through an a priori power analysis. Patients were identified from a nationwide dataset of hospitals participating in the Japanese DPC system between April 2016 and March 2022. During this period, approximately 1,100 hospitals consistently contributed medical records to the DPC system and were approved for inclusion in this study. The analysis included patients who underwent surgical treatment for hip fractures at these hospitals across Japan. This clinical study specifically targeted elderly patients aged 65 years and older with hip fractures, focusing on the incidence of postoperative complications and short-term in-hospital mortality, particularly among those with low BMI (defined as < 17.0 kg/m^2^). Patients in the hip fracture cohort were identified from the registry based on three criteria: (1) the principal diagnosis, (2) the primary reason for hospital admission, and (3) the condition requiring the greatest use of medical resources. Patients younger than 65 years and those managed non-operatively were excluded. All eligible cases meeting these criteria within the study period were analyzed to ensure a comprehensive and representative sample.

### Outcomes of interest

The postoperative complications evaluated in this study included VTE, pneumonia, urinary tract infection, cognitive dysfunction, and in-hospital mortality. Postoperative cognitive dysfunction was identified using ICD-10 codes F010, F011, F012, F019, F03, F107, G238, G300, G301, G308, G309, G310, and G318, which cover cognitive dysfunction and delirium occurring in the postoperative period, as previously described [20]. Each outcome was identified using diagnostic codes. In the DPC database, these diagnoses are typically recorded by the attending physician at the time of discharge and subsequently used for administrative claims. Since mortality is also included as an outcome, the occurrence of outcomes was assessed throughout the entire hospitalization period. Secondary outcomes included the length of hospital stay and volume of perioperative blood transfusion.

### Covariates

Covariates considered in the analysis included age, sex, type of anesthesia, type of hip fracture, Charlson Comorbidity Index, and the presence of comorbidities such as hypertension, diabetes, cerebrovascular disease, chronic renal dysfunction, and cognitive impairment. Hip fractures were categorized using ICD-10 codes: S7200 for femoral neck fractures, S7210 for trochanteric fractures, and S7220 for subtrochanteric fractures.

### Propensity score matching

**Fig. 1:**
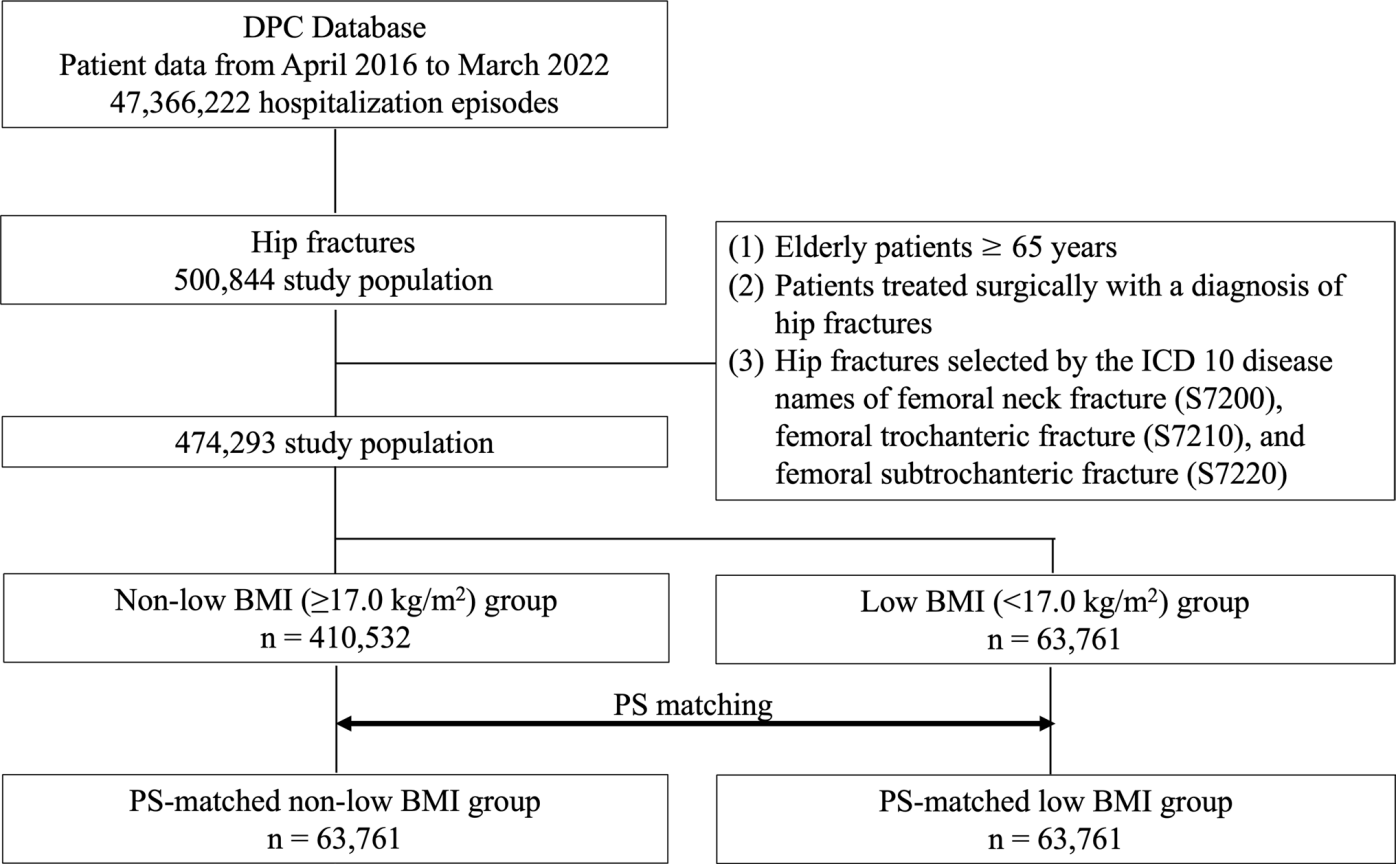
Flow diagram of patient selection for low body mass index (BMI) and non-low BMI patients with hip fracture and propensity score (PS) matching. This diagram shows the method for extracting target patients from the DPC database and PS matching for dialysis and non-dialysis patients with hip fractures

To reduce confounding, we conducted 1:1 propensity score matching using clinically relevant covariates. The propensity score model was constructed via logistic regression and included variables such as age, sex, type of anesthesia, type of hip fracture, surgical procedure, Charlson Comorbidity Index, and major comorbidities (e.g., hypertension, diabetes, cerebrovascular disease, chronic renal dysfunction, and cognitive impairment). Matching was performed with nearest-neighbor without replacement, using a caliper width of 0.2 standard deviations of the logit of the propensity score [32]. Covariate balance was evaluated using standardized mean differences (SMDs), with SMD < 0.1 indicating adequate balance. Independent associations with outcomes were then examined using multivariate logistic regression in the matched cohort, with purposeful selection of variables and model discrimination assessed using the C-statistic. Figure 1 illustrates the patient selection process.

Figure 1 illustrates a schematic overview of the patient selection process. From the dataset covering the period between April 2016 and March 2022, a total of 474,293 patients met the inclusion and exclusion criteria. Of these, 410,532 were classified as non-low BMI and 63,761 as low BMI. Following 1:1 propensity score matching based on age, sex, Charlson Comorbidity Index, use of general anesthesia, comorbidities, type of hip fracture, and surgical procedure, matched cohorts of 63,761 patients each were obtained for the low BMI and non-low BMI groups.

Table 1 presents the baseline characteristics of patients with low and non-low BMI who underwent surgical treatment for hip fractures. Prior to propensity score matching, standardized mean differences (SMDs) exceeded 0.1 for hypertension, diabetes, and cognitive impairment, indicating notable imbalances between the groups. Compared with the non-low BMI group, patients with low BMI had a higher prevalence of cognitive impairment and a lower prevalence of hypertension and diabetes. General anesthesia was commonly used, and femoral neck fractures were the most frequent fracture type in both groups. Following 1:1 propensity score matching, all covariates—including age, sex, Charlson Comorbidity Index, comorbidities, use of general anesthesia, fracture type, and surgical procedure—achieved SMDs below 0.1, indicating adequate balance across matched groups. The C-statistic for the propensity score model was 0.736, reflecting good discriminative ability.

**Table 1:** Characteristics of patients before and after propensity score matching

	Before PS matching			After PS matching		
	Low BMI (<17.0 kg/m^2^)			Low BMI (<17.0 kg/m^2^)		
	(+)	(−)	SMD	(+)	(−)	SMD
n	63,761	410,532		63,761	63,761	
*Sex*						
Male	12,312 (19.3%)	90,852 (22.1%)	0.069	12,312 (19.3%)	12,303 (19.3%)	0.0002
Female	51,449 (80.7%)	319,680 (77.9%)		51,449 (80.7%)	51,458 (80.7%)	
Age	85.2 ± 7.6	84.3 ± 7.7	0.021	85.2 ± 7.6	85.2 ± 7.6	0.003
Charlson comorbidity index	1.2 ± 1.3	1.2 ± 1.3	0.005	1.2 ± 1.3	1.2 ± 1.3	0.017
Hypertension	20,762 (32.6%)	162,050 (39.5%)	0.144	20,762 (32.6%)	20,734 (32.5%)	0.001
Diabetes	7857 (12.3%)	80,010 (19.5%)	0.198	7857 (12.3%)	7738 (12.2%)	0.002
Cerebrovascular disorder	5813 (9.1%)	42,919 (10.5%)	0.045	5813 (9.1%)	5567 (8.7%)	0.013
Chronic renal dysfunction	2800 (4.4%)	22,763 (5.5%)	0.053	2800 (4.4%)	2645 (4.2%)	0.012
Cognitive impairment	16,796 (26.3%)	87,425 (21.3%)	0.104	16,796 (26.3%)	16,638 (26.1%)	0.006
Early surgery (≤ 2 days)	30,691 (48.1%)	196,245 (47.8%)	0.007	30,691 (48.1%)	30,955 (48.6%)	0.008
General anesthesia	37,239 (58.4%)	251,176 (61.2%)	0.057	37,239 (58.4%)	37,369 (58.6%)	0.004
*Fracture classification*						
Femoral neck	32,502 (51.0%)	205,922 (50.2%)	0.027	32,502 (51.0%)	32,662 (51.2%)	0.007
Trochanteric	30,238 (47.4%)	195,403 (47.6%)		30,238 (47.4%)	30,149 (47.3%)	
Subtrochanteric	1021 (1.6%)	9207 (2.2%)		1021 (1.6%)	950 (1.5%)	
*Surgical procedure*						
ORIF	40,674 (63.8%)	257,776 (62.8%)	0.021	40,674 (63.8%)	39,569 (62.1%)	0.036
Hip arthroplasty	23,087 (36.2%)	152,756 (37.2%)		23,087 (36.2%)	24,192 (37.9%)	

One-to-one PS matching was performed

Data is shown as mean ± standard deviation

Early surgery was defined as surgery performed on the day of admission or the following day

*PS* propensity score, *BMI* body mass index, *SMD* standard mean difference, *ORIF* open reduction and internal fixation

### Statistical analyses

Continuous variables were presented as mean ± standard deviation. Comparisons between the low BMI and non-low BMI groups were performed using the χ^2^ test for categorical variables and Student’s t-test for continuous variables. Univariate logistic regression was conducted to assess the association between low BMI and in-hospital complications. Subsequently, multivariate logistic regression analyses were performed for clinically significant complications and in-hospital mortality. To further evaluate the relationship between low BMI and postoperative complications, additional multivariate logistic regression analyses were conducted, adjusting for dialysis status and the covariates used in the propensity score model. As a sensitivity analysis, the BMI threshold for defining low BMI was redefined as < 18.5 kg/m^2^ instead of < 17.0 kg/m^2^. The findings remained consistent with the primary analysis, supporting the robustness of the results. Survival differences between groups were assessed using the log-rank test. Given the large sample size, a conservative significance threshold of p < 0.001 was applied to reduce the risk of Type I error. All statistical tests were two-sided. Although multiple outcomes were evaluated (mortality, cognitive dysfunction, VTE, and pneumonia), no formal adjustment for multiple comparisons was applied, as each outcome was predefined and considered of equal clinical relevance. All statistical analyses were performed using JMP version 18 (SAS Institute, Cary, NC, USA).

## Results

Supplementary Table 1 and Table 2 summarize postoperative complication rates. Before matching, VTE was less frequent in the low-BMI group, whereas urinary tract infection, pneumonia, and in-hospital mortality occurred more often (Supplementary Table 1). After matching, these trends persisted, with a markedly higher in-hospital mortality rate in the low-BMI group (3.4% vs. 1.6%) (Table 2).

**Table 2:** Comparison of complications after propensity score matching

	After PS matching		
	Low BMI (<17.0 kg/m^2^)		
	(+)	(−)	*p*-value
Venous thromboembolism	2487 (3.9%)	2888 (4.5%)	< 0.0001*
Urinary tract infection	2314 (3.6%)	1930 (3.0%)	< 0.0001*
Pneumonia	3391 (5.3%)	1890 (3.0%)	< 0.0001*
Cognitive dysfunction	940 (1.5%)	838 (1.3%)	0.015
In-hospital mortality	2153 (3.4%)	988 (1.6%)	< 0.0001*
Length of hospitalization (days)	35.8 ± 29.0	35.3 ± 27.8	0.0018
Blood transfusion Day 0 (unit)	0.52 ± 1.10	0.43 ± 1.06	< 0.0001*
Blood transfusion Day 1 (unit)	0.38 ± 0.90	0.31 ± 0.83	< 0.0001*

One-to-one PS matching was performed

*PS* propensity score, *BMI* body mass index

^*^p-values of < 0.001 are considered significant by the χ^2^ test and Student’s t-test

Table 3 shows the regression analyses. In the matched cohort, low BMI remained independently associated with urinary tract infection (OR 1.203, 95% CI 1.131–1.280), pneumonia (OR 1.850, 95% CI 1.746–1.961), and in-hospital mortality (OR 2.233, 95% CI 2.068–2.411; all p < 0.0001).

**Table 3:** Univariate and multivariate logistic regression analyses of postoperative complications associated with low BMI after propensity score matching

	Univariate		Multivariate	
	Odds Ratio (95% CI)	*p*-value	Odds Ratio (95% CI)	*p*-value
Urinary tract infection	1.206 (1.134–1.283)	< 0.0001*	1.203 (1.131–1.280)	< 0.0001*
Pneumonia	1.839 (1.736–1.947)	< 0.0001*	1.850 (1.746–1.961)	< 0.0001*
In-hospital mortality	2.220 (2.057–2.396)	< 0.0001*	2.233 (2.068–2.411)	< 0.0001*

*CI* confidence interval

^*^p-values of < 0.001 are considered significant by the χ^2^ test

Sex-stratified analyses (Supplementary Table 2) revealed consistent associations in both sexes, with particularly pronounced differences among males (pneumonia 11.1% vs. 6.1%; mortality 6.9% vs. 3.0%). In contrast, UTI was significantly more frequent only among females with low BMI.

Table 4 presents the results of the sensitivity analysis. When redefining low BMI as < 18.5 kg/m^2^, a total of 126,828 patients met this criterion. Using the same methodology as the main analysis, 1:1 propensity score matching was performed, yielding 126,812 matched pairs in the low BMI and non-low BMI groups. Sensitivity analysis using a BMI cutoff of < 18.5 kg/m^2^ yielded consistent results.

**Table 4:** Sensitivity analysis of postoperative complications by two definitions of low BMI (< 18.5 vs < 17.0 kg/m^2^) after PS matching

	Low BMI (<18.5 kg/m^2^)			Low BMI (<17.0 kg/m^2^)		
	(+)	(−)	*p*-value	(+)	(−)	*p*-value
n	126,812	126,812		63,761	63,761	
Venous thromboembolism	5157 (4.1%)	6091 (4.8%)	< 0.0001*	2487 (3.9%)	2888 (4.5%)	< 0.0001*
Urinary tract infection	4407 (3.5%)	3773 (3.0%)	< 0.0001*	2314 (3.6%)	1930 (3.0%)	< 0.0001*
Pneumonia	5795 (4.6%)	3459 (2.7%)	< 0.0001*	3391 (5.3%)	1890 (3.0%)	< 0.0001*
Cognitive dysfunction	1846 (1.5%)	1722 (1.4%)	0.037	940 (1.5%)	838 (1.3%)	0.015
In-hospital mortality	3534 (2.8%)	1824 (1.4%)	< 0.0001*	2153 (3.4%)	988 (1.6%)	< 0.0001*
Length of hospitalization (days)	35.5 ± 28.0	35.2 ± 27.2	0.021	35.8 ± 29.0	35.3 ± 27.8	0.0018
Blood transfusion Day 0 (unit)	0.50 ± 1.10	0.42 ± 1.04	< 0.0001	0.52 ± 1.10	0.43 ± 1.06	< 0.0001*
Blood transfusion Day 1 (unit)	0.37 ± 0.89	0.30 ± 0.81	< 0.0001	0.38 ± 0.90	0.31 ± 0.83	< 0.0001*

One-to-one PS matching was performed

^*^p-values of < 0.001 are considered significant by the χ^2^ test and Student’s t-test

*PS* propensity score, *BMI* body mass index

**Fig. 2:**
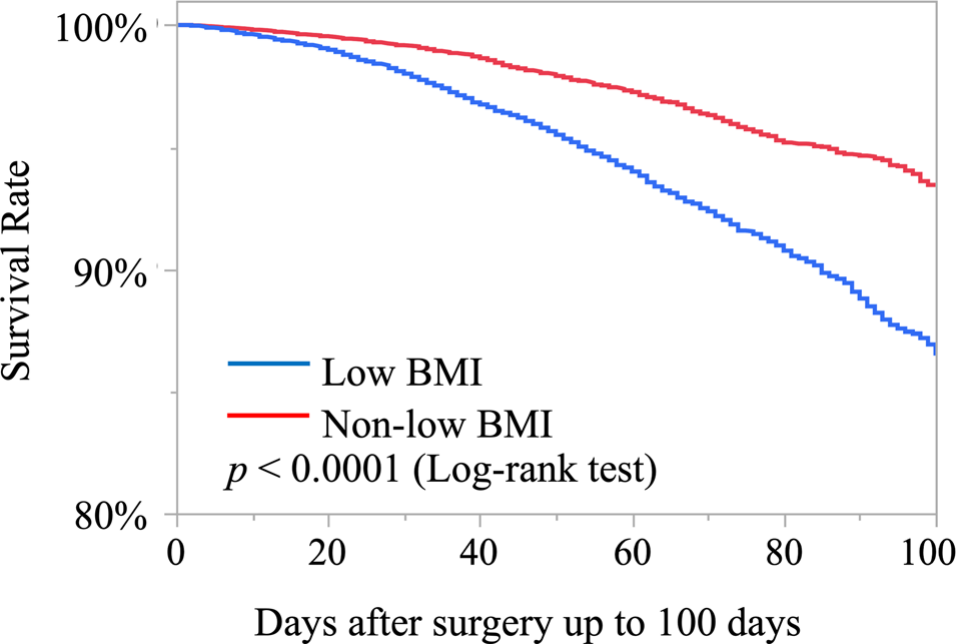
Kaplan–Meier survival curves for low body mass index (BMI) and non-low BMI groups. The number of patients at risk is shown below each curve at predefined time points. The 30-day survival rate was 98.0% in the low BMI group and 99.1% in the non-low BMI group (p < 0.0001, log-rank test)

Figure 2 illustrates survival curves. Most in-hospital deaths occurred within 100 days after surgery; therefore, curves were presented up to 100 days. The 30-day survival rate was 98.0% in the low-BMI group and 99.1% in the non–low BMI group, with consistently lower survival in the low-BMI group throughout hospitalization (log-rank p < 0.0001).

## Discussion

In this nationwide DPC database study of over 470,000 elderly patients with hip fractures who underwent surgical treatment, we demonstrated that low BMI was associated with significantly higher risks of postoperative complications and in-hospital mortality. Specifically, after rigorous propensity score matching, the risks of urinary tract infection, pneumonia, and in-hospital mortality remained significantly elevated in the low-BMI group, underscoring the robustness of our findings. In addition, low BMI patients required greater volumes of perioperative blood transfusion both on the day of surgery and on the following day, and their survival curves showed persistently lower in-hospital survival. Although hospital stay was initially longer in the low-BMI group, this difference disappeared after matching. This finding suggests that the poor prognosis in these patients is mainly attributable to infection- and mortality-related risks, rather than to prolonged hospitalization itself.

Our results are consistent with previous epidemiological studies demonstrating that underweight status predisposes to osteoporosis, fracture occurrence, postoperative complications, transfusion, and mortality [9–16]. However, this study extends the existing evidence by showing, in one of the largest hip fracture cohorts to date, that low BMI is independently associated with increased perioperative infection rates and mortality even after careful adjustment for comorbidities and surgical factors. Importantly, these associations remained robust in sensitivity analyses using the WHO cutoff of BMI < 18.5 kg/m^2^, highlighting that the observed risks are not dependent on a specific threshold.

Several mechanisms may explain the increased incidence of pneumonia and urinary tract infection in the low BMI group. First, our cohort demonstrated a higher prevalence of cognitive impairment among patients with low BMI, a factor that may contribute to immobility, impaired swallowing, and increased risk of aspiration pneumonia [33, 34]. Second, frailty and sarcopenia, which are common in underweight individuals, likely predispose them to urinary stasis and catheter-related infections [35, 36]. Third, nutritional deficiencies and reduced immune competence in the low BMI population may impair host defense mechanisms, making these patients more vulnerable to postoperative infections [28, 29, 37]. The significantly greater perioperative transfusion requirements observed in our study further suggest that low BMI patients may have reduced physiological reserves or baseline anemia, both of which could exacerbate postoperative vulnerability [30].

With respect to mortality, our data showed that the in-hospital death rate was more than twice as high in the low BMI group compared with the non-low BMI group. Although the specific causes of death could not be determined from this database, it is reasonable to hypothesize that the excess mortality reflects an interplay of factors. Higher rates of pneumonia and urinary tract infection may directly contribute to mortality through sepsis and respiratory failure [38–40]. In addition, frailty, poor nutritional status, reduced cardiopulmonary reserve, and perioperative instability—as suggested by increased transfusion requirements—may amplify the impact of surgical stress and accelerate decompensation of underlying comorbidities [41, 42]. Overall, these findings suggest that low BMI represents not only reduced body composition but also a surrogate marker of multiple vulnerabilities that markedly increase perioperative mortality risk.

The clinical implications of these findings are significant. Our results indicate that elderly hip fracture patients with low BMI represent a particularly high-risk subgroup, warranting targeted perioperative management. Preoperative nutritional assessment, perioperative infection-prevention strategies, respiratory rehabilitation, and early mobilization should be prioritized in this population [43, 44].

This study has several limitations. First, the analysis was based on an administrative claims database, which may be subject to inaccuracies in diagnostic coding. Second, as the study period extended from 2016 to 2022, temporal factors such as evolving clinical practices and the COVID-19 pandemic may have influenced the results. However, in a prior nationwide database study [27], postoperative complications of hip fracture patients were compared before and after the COVID-19 pandemic. That analysis demonstrated that pneumonia risk was not specifically increased by COVID-19 pneumonia when considered separately. Therefore, the elevated pneumonia risk in low-BMI patients observed in the present study is unlikely to be solely attributable to the pandemic period. In addition, detailed clinical parameters such as nutritional markers, bone mineral density, laboratory data, and perioperative management strategies were not available, potentially leaving residual confounding. Third, while propensity score matching and sensitivity analyses minimized bias, unmeasured confounding factors cannot be excluded. Fourth, although the DPC system ensures standardized nationwide data capture, the accuracy of diagnostic coding may vary among institutions, and misclassification cannot be entirely excluded. Such misclassification could lead to either overestimation or underestimation of the association between low BMI and postoperative outcomes. Fifth, the study population included only patients with hip fractures who were treated in acute care hospitals and reported in the DPC data system. This study did not include patients in non-DPC-reported beds (approximately 30% of general hospital beds) or those not treated in acute care hospitals [45]. Finally, as our data were derived from a Japanese nationwide database, the generalizability of these results to other populations with different demographic or nutritional profiles remains uncertain.

In conclusion, this large-scale nationwide study demonstrates that low BMI is an independent risk factor for postoperative infections, greater transfusion requirements, and in-hospital mortality in elderly patients undergoing hip fracture surgery. Recognition of low BMI as a prognostic marker underscores the importance of incorporating nutritional and frailty assessments into perioperative care pathways to improve outcomes in this vulnerable patient population.

## Supplementary Information

Below is the link to the electronic supplementary material.

Supplementary file1 (DOCX 37 KB)

## Data Availability

The datasets used and/or analysed during the current study are available from the corresponding author on reasonable request.

## Abbreviations

BMI: Body mass index
CI: Confidence interval
DPC: Diagnosis procedure combination
OR: Odds ratio
VTE: Venous thromboembolism
