# Supplementary material for: Low BMI and postoperative outcomes in elderly hip fracture patients: a Japanese nationwide database study
Source: J Bone Miner Metab. 2025 Nov 25;44(1):97–105. doi: 10.1007/s00774-025-01660-5 (PMC12890968; doi:10.1007/s00774-025-01660-5)
Supplement: Supplementary file 1 — Supplementary file1 (DOCX 37 KB) [file 774_2025_1660_MOESM1_ESM.docx]

Supplementary Table 1. Comparison of complications before propensity score matching

|  | Before PS matching | | |
| --- | --- | --- | --- |
|  | Low BMI (<17.0 kg/m^2^) | | |
|  | (+) | (−) | *p*-value |
| Venous thromboembolism | 2487 (3.9%) | 19170 (4.7%) | < 0.0001* |
| Urinary tract infection | 2314 (3.6%) | 12202 (3.0%) | < 0.0001* |
| Pneumonia | 3391 (5.3%) | 11704 (2.9%) | < 0.0001* |
| Cognitive dysfunction | 940 (1.5%) | 5782 (1.4%) | 0.19 |
| In-hospital mortality | 2153 (3.4%) | 6356 (1.6%) | < 0.0001* |
| Length of hospitalization (days) | 35.8 ± 29.0 | 35.3 ± 27.9 | 0.0003* |
| Blood transfusion Day 0 (unit) | 0.52 ± 1.10 | 0.44 ± 1.06 | < 0.0001* |
| Blood transfusion Day 1 (unit) | 0.38 ± 0.90 | 0.30 ± 0.82 | < 0.0001* |
| One-to-one PS matching was performed. | | | |
| **p*-values of < 0.001 are considered significant by the χ^2^ test and Student’s t-test; PS means propensity score; BMI means body mass index. | | | |

Supplementary Table 2. Sex-stratified postoperative outcomes in propensity score–matched hip fracture patients with and without low BMI

|  | Female | | | Male | | |
| --- | --- | --- | --- | --- | --- | --- |
|  | Low BMI (+) | Low BMI (−) | *p*-value | Low BMI (+) | Low BMI (−) | *p*-value |
| n | 51449 | 51458 |  | 12312 | 12303 |  |
| Venous thromboembolism | 2098 (4.1%) | 2458 (4.8%) | < 0.0001* | 389 (3.2%) | 430 (3.5%) | 0.14 |
| Urinary tract infection | 1964 (3.8%) | 1570 (3.1%) | < 0.0001* | 350 (2.8%) | 360 (2.9%) | 0.69 |
| Pneumonia | 2025 (3.9%) | 1144 (2.2%) | < 0.0001* | 1366 (11.1%) | 746 (6.1%) | < 0.0001* |
| Cognitive dysfunction | 748 (1.5%) | 662 (1.3%) | 0.021 | 192 (1.6%) | 176 (1.4%) | 0.41 |
| In-hospital mortality | 1303 (2.5%) | 625 (1.2%) | < 0.0001* | 850 (6.9%) | 363 (3.0%) | < 0.0001* |
| Length of hospitalization (days) | 35.4 ± 28.9 | 35.1 ± 27.8 | 0.052 | 37.3 ± 29.5 | 36.1 ± 27.8 | 0.002 |
| Blood transfusion Day 0 (unit) | 0.52 ± 1.10 | 0.44 ± 1.06 | < 0.0001 | 0.51 ± 1.14 | 0.39 ± 1.05 | < 0.0001* |
| Blood transfusion Day 1 (unit) | 0.39 ± 0.91 | 0.32 ± 0.84 | < 0.0001 | 0.35 ± 0.88 | 0.27 ± 0.81 | < 0.0001* |
| One-to-one PS matching was performed. | | | | | | |
| **p*-values of < 0.001 are considered significant by the χ^2^ test and Student’s t-test; BMI means body mass index. | | | | | | |
